# Characteristics and Mechanism of Vinyl Ether Cationic Polymerization in Aqueous Media Initiated by Alcohol/B(C_6_F_5_)_3_/Et_2_O

**DOI:** 10.3390/polym11030500

**Published:** 2019-03-14

**Authors:** Jinghan Zhang, Yibo Wu, Kaixuan Chen, Min Zhang, Liangfa Gong, Dan Yang, Shuxin Li, Wenli Guo

**Affiliations:** 1 College of Material Science and Engineering, Beijing University of Chemical Technology, Beijing 100029, China; zhangjinghan0517@163.com; 2 Department of Materials Science and Engineering, Beijing Institute of Petrochemical Technology, Beijing 102617, China; sunyongjun1120@163.com (K.C.); zm0127@nwpu.edu.cn (M.Z.); gongliangfa@bipt.edu.cn (L.G.); yangdan@bipt.edu.cn (D.Y.); lishuxin@bipt.edu.cn (S.L.); 3 Beijing Key Lab of Special Elastomeric Composite Materials, Beijing 102617, China

**Keywords:** aqueous cationic polymerization, suspension polymerization, emulsion polymerization, poly(vinyl ether), tris(pentafluorophenyl)borane, density functional theory

## Abstract

Aqueous cationic polymerizations of vinyl ethers (isobutyl vinyl ether (IBVE), 2-chloroethyl vinyl ether (CEVE), and *n*-butyl vinyl ether (*n*-BVE)) were performed for the first time by a CumOH/B(C_6_F_5_)_3_/Et_2_O initiating system in an air atmosphere. The polymerization proceeded in a reproducible manner through the careful design of experimental conditions (adding initiator, co-solvents, and surfactant or decreasing the reaction temperature), and the polymerization characteristics were systematically tested and compared in the suspension and emulsion. The significant difference with traditional cationic polymerization is that the polymerization rate in aqueous media using B(C_6_F_5_)_3_/Et_2_O as a co-initiator decreases when the temperature is lowered. The polymerization sites are located on the monomer/water surface. Density functional theory (DFT) was applied to investigate the competition between H_2_O and alcohol combined with B(C_6_F_5_)_3_ for providing a theoretical basis. The effectiveness of the proposed mechanism for the aqueous cationic polymerization of vinyl ethers using CumOH/B(C_6_F_5_)_3_/Et_2_O was confirmed.

## 1. Introduction

Conventional cationic polymerizations of vinyl monomers, such as isobutylene (IB), vinyl ethers, and styrene, are usually achieved by using initiator/co-initiator systems primarily in chlorinated solvents under low-temperature and strict water-free conditions [1,2,3,4,5,6,7,8]. Chlorinated solvents seriously damage the environment, and their applications should be limited [9,10]. Under the advocacy of green chemistry, the use of environmentally benign solvents in cationic polymerization has been extensively studied, such as using fewer toxic solvents (e.g., toluene or *n*-hexane) [11,12,13], supercritical CO_2_ [14,15], and ionic liquids [16,17,18,19,20]. Water is one of the most ideal polymerization solvents. However, traditional co-initiators are water-sensitive and decompose when excessive quantities of water are present [21,22]. Because Yb(OTf)_3_ was first investigated as a water-tolerant co-initiator for its ability to induce the polymerization of *p*-methoxystyrene (pMOS) in 1999 [23], considerable attention has been paid to the utilization of aqueous cationic polymerization. With the development of various water-tolerant Lewis acids (LAs), this new polymer synthesis technology has achieved remarkable breakthroughs [21].

Early studies on aqueous cationic polymerizations of reactive monomer *p*-methoxystyrene using 1-chloro-1-(p-methoxyphenyl)ethane (pMOS-HCl) combined with lanthanide triflates as an initiator/co-initiator system shared two main features: (a) the polymerization sites were located on the monomer/water surface; (b) the molecular weights of the polymers obtained in aqueous media were relatively low due to the “critical degree of polymerization” effect [23,24,25]. The next generation of catalysts, called “Lewis acid surfactant combined catalysts” (LASC), that is, a triflic acid in conjunction with a non-ionic surfactant, can transfer the polymerization site from the monomer/water surface to the interior of the monomer, resulting in the production of long polymer chains [26,27,28,29]. However, these LASCs still lack activity, and the polymerizations proceed at high temperatures (from 40 °C to 60 °C). Recently, potential initiating systems based on boranes were introduced for the aqueous cationic polymerization of industrially important monomers. BF_3_OEt_2_ induces the styrene cationic polymerizations when a small amount of water is present [30,31]. However, excessive quantities of water would easily deactivate this co-initiator in styrene polymerization [30]. B(C_6_F_5_)_3_, which does not decompose in an air atmosphere or in water, successfully promotes the aqueous cationic polymerization of styrene and its derivatives [32,33], cyclopentadiene [33,34] and isoprene [35], but this material is inactive for polymerizing IB [36] and isobutyl vinyl ether (IBVE) [37] in aqueous media. The noncommercial chelating diborane (o-C_6_F_4_[B(C_6_F_5_)_2_]_2_) [38] and diborole (o-C_6_F_4_[9-BC_12_F_8_]_2_) [38,39] ionize various cumyl derivatives and play the role of co-initiators in the aqueous cationic polymerization of IB. (Pentafluorophenyl)group 13 metal compounds Al(C_6_F_5_)_3_ and Ga(C_6_F_5_)_3_ [36], which are structurally similar to B(C_6_F_5_)_3_ but unstable in an air atmosphere, were recently tested for the aqueous cationic polymerization of IB under harsh experimental conditions (in LiCl/NaCl/H_2_O diluents under N_2_ atmosphere at −60 °C).

To date, only IBVE has been reported for cationic polymerization of vinyl ethers in aqueous media, and there have been no reports on the aqueous cationic polymerization of 2-chloroethyl vinyl ether (CEVE) and *n*-butyl vinyl ether (*n*-BVE). To the best of our knowledge, only two types of co-initiators, namely, BF_3_OEt_2_ and H_3_PW_12_O_40_ and its salts have been reported for cationic polymerization of IBVE in aqueous media [22,37]. BF_3_OEt_2_ induces polymerization in a highly exothermic manner, and H_3_PW_12_O_40_ and its salts induce polymerization in an N_2_ atmosphere with a previous preparation of IBVE–HCl adduct to provide low-molecular-weight poly(IBVE)s (1200–4500 g mol^-1^). It is generally believed that B(C_6_F_5_)_3_, which is used to initiate the aqueous cationic polymerization of styrene, cannot initiate the aqueous cationic polymerization of IBVE [37]. However, we believe that B(C_6_F_5_)_3_, which has high activity, will promote the aqueous cationic polymerization of more active monomer vinyl ethers. In this study, we synthesized poly(IBVE)s, poly(CEVE), and poly(*n*-BVE) in aqueous suspensions and emulsions by using B(C_6_F_5_)_3_/Et_2_O complexes under an air atmosphere in the range of −10 °C to 20 °C. Severely exothermic reactions and poor polymerization reproducibility were overcome by identifying an appropriate initiator, diluents, and surfactants. We observed that the polymerization rate decreased when the temperature was reduced. The polymerization sites were located on the monomer/water surface. The possible mechanism of vinyl ether cationic polymerization in aqueous media was systematically studied by density functional theory (DFT), kinetics, and end group analysis.

## 2. Materials and Methods 

### 2.1. Materials

Methanol (Beijing Chemical Works, Beijing, China, 99.9%), ethanol (EtOH, Beijing Chemical Works, 99.7%), cumyl alcohol (CumOH, Beijing Chemical Works, 97%), diethyl ether (Beijing Chemical Works, 99.5%), isopropanol (IPA, Beijing Chemical Works, 99.5%), B(C_6_F_5_)_3_ (J&K Scientific Ltd., Beijing, China, 97%), hexadecyltrimethylammonium bromide (CTAB, J&K Scientific Ltd., 99%), toluene (Beijing Chemical Works, 99.5%), 4-nonylphenyl-polyethylene glycol (NP-40, J&K Scientific Ltd.), NaCl (Beijing Chemical Works, 99%), sodium dodecylbenzenesulfonate (SDBS, J&K Scientific Ltd., 95%), deuterated chloroform (CDCl_3_, J&K Scientific Ltd., 99.8%), *n*-hexane (Beijing Chemical Works, 99.5%), and tetrahydrofuran (THF) (J&K Scientific Ltd., 99.9%) were used as received. IBVE (J&K Scientific Ltd., 99.0%), 2-chloroethyl vinyl ether (J&K Scientific Ltd., 97.0%), and *n*-butyl vinyl ether (J&K Scientific Ltd., 98.0%) were distilled in a nitrogen atmosphere before use.

### 2.2. States of Suspension and Emulsion

Aqueous suspension and emulsion systems were investigated in this study. For the suspension, high-purity water, NaCl solution, and a mixture of water and toluene or *n*-hexane were used as polymerization media. Monomer droplets were visible to the naked eye at 150 rpm (mechanical stirring). Organic and water phases were separated rapidly after stopping the mechanical stirrer. For the emulsion, CTAB, NP-40, and SDBS were applied to the media. Phase separation was observed at 0.5 h after stopping the mechanical stirrer (150 rpm). The average particle size ranged from 51 to 59 nm, and the standard deviation ranged from 17 to 20 nm. B(C_6_F_5_)_3_ did not dissolve when it was added to the high-purity water. After adding a small amount of diethyl ether, B(C_6_F_5_)_3_ dissolved and formed a milky solution.

### 2.3. Polymerizations

#### 2.3.1. Vinyl Ether Cationic Polymerization in Suspension

The polymerizations were implemented in an air atmosphere in the range between −10 °C and 20 °C in glass culture tubes (60 mL). H_2_O (3 mL), the monomer (1 mL), and an initiator (EtOH, IPA, or CumOH) were added to a reactor, which was then warmed to a predetermined polymerization temperature for 20 min by immersion in an ethanol/water (50/50) bath. In several cases, NaCl, *n*-hexane, and toluene were added to the recipe. Then, B(C_6_F_5_)_3_ (0.128 g, 2.5 × 10^−4^ mol), H_2_O (2 mL), and diethyl ether (5×10^−4^ mol) were mixed in a beaker in order and warmed to a predetermined polymerization temperature for a sufficient amount of time. Then, the mixture in the beaker was poured into the reactor under mechanical stirring at 150 rpm. The temperature probe (Testo 176T4) was located below the liquid level. After a predetermined time, the suspension media was poured out into excess methanol. The polymers were washed three times with methanol and dried in a vacuum oven at 30 °C.

#### 2.3.2. Vinyl Ether Cationic Polymerization in Emulsion

The polymerizations were implemented under the condition of adding an emulsifier. H_2_O (3 mL), a surfactant (NP-40, CTAB, or SDBS), monomer (1 mL), and CumOH (0.034 g, 2.5×10^−4^ mol) were added to a reactor with mechanical stirring at 150 rpm. The reactor was then warmed to a predetermined polymerization temperature for 20 min by immersion in an ethanol/water (50/50) bath. B(C_6_F_5_)_3_ (0.128 g, 2.5 × 10^−4^ mol), H_2_O (2 mL, 0.13 M), and diethyl ether (5 × 10^−4^ mol) were mixed in a beaker in order and warmed to a predetermined polymerization temperature for a sufficient amount of time. The mixture in the beaker was poured afterward into the reactor. Subsequent processing was similar to that for polymerization in an aqueous suspension.

### 2.4. Measurements

Proton nuclear magnetic resonance (^1^H-NMR) spectra of the poly(vinyl ether)s were recorded in CDCl_3_ as solvents at 25 °C on a Bruker-500 MHz spectrometer calibrated with tetramethylsilane as the internal standard (δ_H_ = 0.00). Molecular weights and *MWD* (*M*_w_*/M*_n_) of the poly(vinyl ether)s were obtained from a gel permeation chromatography (GPC) system with universal calibration using a Waters e2695 separations module, a Waters 2489 UV detector, a Waters 2414 RI detector, and four Waters styragel columns connected in the following order: 500, 10^3^, 10^4^, and 10^5^. THF served as a solvent for the polymers with a concentration of 20 mg of polymer/10 mL of THF and mobile phase at a flow rate of 1.0 mL/min. ATR-FTIR spectra were recorded in situ by using a Mettler Toledo ReactIR 15 instrument with a DiComp probe coupled with an Material HgCdTe detector via AgX fiber. Sampling wavenumbers were from 4000 to 600 cm^−1^ at a resolution of 2 cm^−1^. The particle size in the emulsion was tested by a dynamic light scattering (DLS) analysis system using a Nanosight LM20. The reaction system temperature was detected with Testo 176T4, a data logger that records temperature per second.

### 2.5. Computational Method

All the calculations were performed with Gaussian 03 software (Version 6.0, Gaussian Inc., Wallingford, CT, USA). Geometry optimizations and vibrational frequency calculations were carried out via the density functional theory (DFT) BP86: Becke’s 1988 exchange functional [40] with Perdew’s correlation functional [41] using 6-31G∗ basis sets for C, H, O, B, F. This gave the charges of carbocation and the atoms’ distances.

## 3. Results and Discussion

### 3.1. Testing of Different Initiators

**Figure 1:**
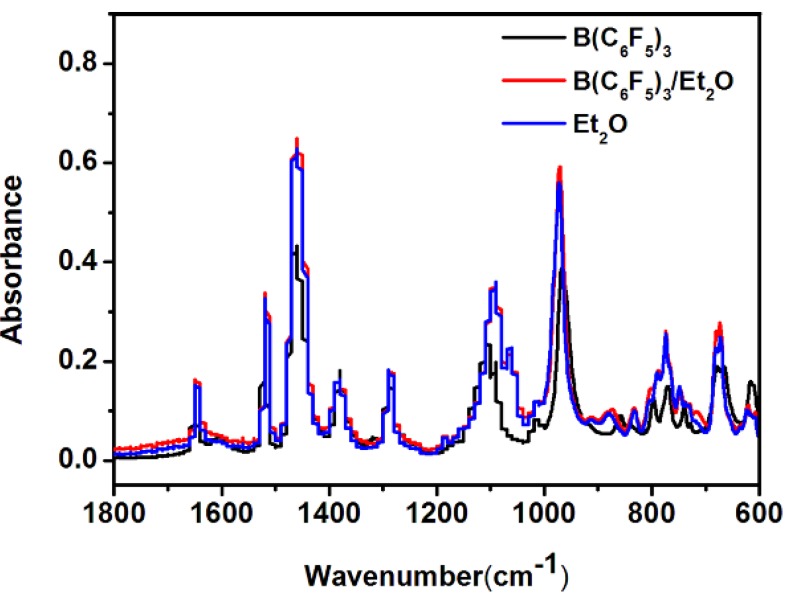
ATR-FTIR spectra for the interaction between B(C_6_F_5_)_3_ and Et_2_O at 20 °C. [B(C_6_F_5_)_3_] = 0.05 M; [Et_2_O] = 0.1 M.

**Figure 2:**
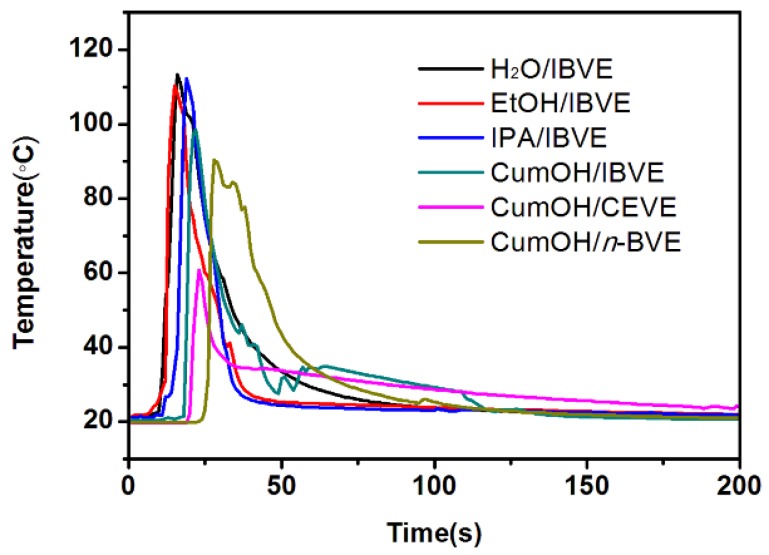
Temperature during cationic polymerizations of vinyl ethers initiated by different alcohols/B(C_6_F_5_)_3_/Et_2_O in aqueous suspensions at 20 °C: [IBVE] = 1.6 M; [CEVE] = 2.0 M; [*n*-BVE] = 1.6 M; [EtOH] = [IPA] = [CumOH] = 0.05 M; [B(C_6_F_5_)_3_] = 0.05 M; [Et_2_O] = 0.1 M. CEVE: 2-chloroethyl vinyl ether; *n*-BVE: *n*-butyl vinyl ether; IPA: isopropanol.

The reaction between B(C_6_F_5_)_3_ and Et_2_O were investigated with ATR-FTIR spectroscopy, as shown in Figure 1. When Et_2_O comes in direct contact with B(C_6_F_5_)_3_, the absorbance at 1091 and 774 cm^-1^ is changed, which indicates the interaction between B(C_6_F_5_)_3_ and Et_2_O [42,43,44]. The B(C_6_F_5_)_3_/Et_2_O complex can react with H_2_O or alcohols to form an active species, which can initiate polymerization (Table 1). Given that IBVE is one of the most reactive monomers in conventional cationic polymerization [16,37], H_2_O as an initiator combined with the co-initiator B(C_6_F_5_)_3_ induced rapid cationic polymerization of IBVE in the aqueous suspension in a highly exothermic (even explosive) and non-reproducible manner at 20 °C (run 1 in Table 1). To achieve an efficient and stable polymerization process, we used primary, secondary, and tertiary alcohols as initiators in this study. The maximum temperatures during polymerizations initiated by H_2_O, EtOH, IPA, and CumOH/B(C_6_F_5_)_3_/Et_2_O were typically above the boiling point of IBVE (83 °C) (Figure 2). Only CumOH/B(C_6_F_5_)_3_ did not induce the polymerization in an explosive and non-reproducible manner (Table 1). The longest preparation time (18 s) and lowest maximum temperature (98.6 °C) during polymerization initiated by CumOH/B(C_6_F_5_)_3_ indicated that using the CumOH/B(C_6_F_5_)_3_ initiating system was the most stable approach. CumOH had high initiating activity in aqueous media, as proven by the polymerization rates of styrene in the aqueous suspension initiated by H_2_O, EtOH, IPA, and CumOH/B(C_6_F_5_)_3_ (Figure S1). Because B(C_6_F_5_)_3_ is dissolved in water before it is placed into the reactor, competitive complexing should occur between H_2_O/B(C_6_F_5_)_3_ and CumOH/B(C_6_F_5_)_3_ initiating systems. DFT was used to study the interactions between the initiator and co-initiator (see Section 3.5 for details). The polymers obtained from the CumOH/B(C_6_F_5_)_3_ initiating system exhibited generally narrower *MWD*s than did those obtained from other systems (Table 1).

### 3.2. Polymerization in Aqueous Suspension

#### 3.2.1. Effect of Initiator Concentration

**Figure 3:**
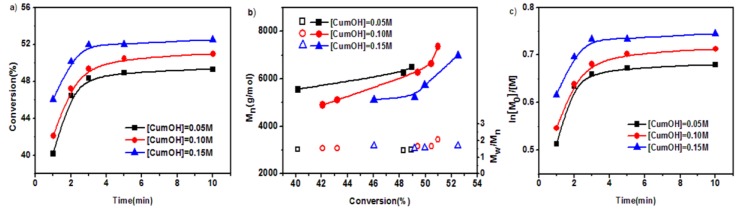
IBVE cationic polymerization initiated by CumOH/B(C_6_F_5_)_3_/Et_2_O in H_2_O at 20 °C: (**a**) conversion vs time; (**b**) *M_n_* and *M_w_*/*M_n_* vs conversion; (**c**) ln[*M*_0_]/[*M*] vs time. [IBVE] = 1.6 M; [B(C_6_F_5_)_3_] = 0.05 M; [Et_2_O] = 0.1 M.

The aqueous cationic polymerization of IBVE was conducted in H_2_O using B(C_6_F_5_)_3_ combined with different concentrations of CumOH at 20 °C (Figure 3). Because B(C_6_F_5_)_3_ was dissolved in water, the polymerization sites in H_2_O were on the monomer/water surface. Figure 3 indicates that the IBVE polymerization rate increased slightly with increases in the concentration of CumOH. The highest monomer conversion was only 52% at [CumOH] = 0.15M (Figure 3a), which may be due to the chain-transfer reaction during polymerization. By contrast, the number average molecular weight (*M_n_*) decreased as the concentration of CumOH increased (Figure 3b). The increase of *M_n_* with monomer conversion at the later stage of polymerization was due to a coupling reaction during the later stages of the polymerization (see Section 3.6 for details). The polymers obtained in H_2_O exhibited broad *MWD*s, indicating that the suspension polymerization was not controlled. The nonlinear curves of ln([*M*_0_]/[*M*]) versus time (Figure 3c) illustrated that the number of active species in aqueous suspension processes decreased continuously with time.

#### 3.2.2. Effect of Diluents

**Figure 4:**
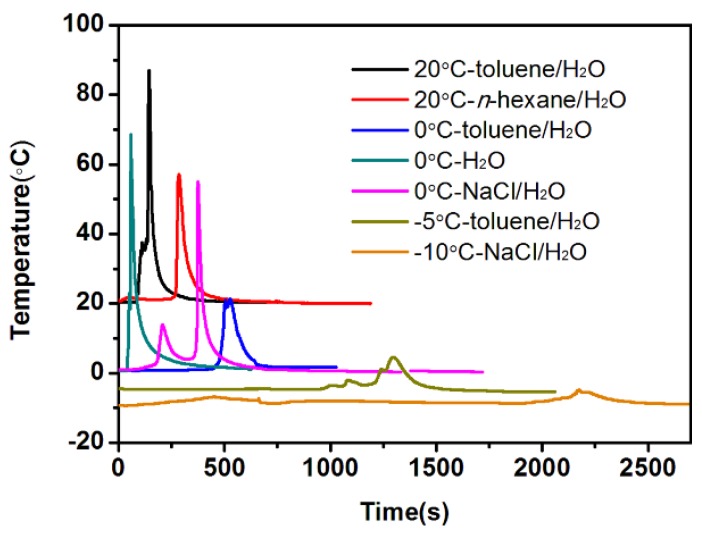
Temperature during IBVE cationic polymerizations initiated by CumOH/B(C_6_F_5_)_3_/Et_2_O in an aqueous suspension: toluene: 1 mL; NaCl: 1 g; [IBVE] = 1.6 M; [CumOH] = [B(C_6_F_5_)_3_] = 0.05 M; [Et_2_O] = 0.1 M.

Given that the polymerization sites in H_2_O were on the monomer/water surface, we assumed that the use of an organic, hardly water-soluble cosolvent or NaCl solution would dilute the monomer and decrease the reaction exotherm. In the presence of *n*-hexane, toluene, or NaCl, polymerization in the aqueous suspension proceeded in a reasonable, reproducible manner from −10 °C to 20 °C. The reaction exotherms decreased, and the preparation times during polymerizations increased in these cases (Figure 4). The use of these diluents (*n*-hexane/H_2_O, toluene/H_2_O, or NaCl solution) led to a decline in monomer conversion and *M_n_*, except for the polymerization in H_2_O at +0 °C (Table 2).

These observations are consistent with the characteristics of interfacial reactions of IBVE polymerizations in aqueous media [37]. The use of co-solvents makes it difficult for B(C_6_F_5_)_3_, which is dissolved in water, to reach the monomer, leading to a low monomer conversion and *M*_n_. In the NaCl solution, the Cl^−^ inhibits the positive charge of the active species [16,17], which results in a decrease in the polymerization rate on the monomer/water surface [45]. The yield and molecular weight of the polymerization in H_2_O at +0 °C (run 6 in Table 2) were lower than those of the polymerizations in other diluents probably because the high-purity water system was extremely near the freezing point.

#### 3.2.3. Effect of Temperature

To increase the monomer conversion and *M_n_*, the reaction temperature of IBVE polymerization in the aqueous suspension was reduced from 20 °C to −10 °C (Table 2). When the reaction temperature was reduced, the reaction exotherms decreased, and the preparation times increased in all suspension processes. Meanwhile, a trend contrary to traditional cationic polymerizations [1,2,3,46] was observed. The yields and *M_n_* obtained in the aqueous suspension processes decreased when the reaction temperature was lowered. The possible reason for these observations might be the loss of the co-initiator activity when reaction temperature decreases. We also examined styrene polymerization in the aqueous suspension (Figure S2), and the characteristics were fully consistent with those in IBVE polymerization in the aqueous suspension. The loss of initiating activity with decreasing temperature might be the real reason B(C_6_F_5_)_3_ could not induce IB cationic polymerization below −20 °C.

#### 3.2.4. Other Vinyl Ethers

**Figure 5:**
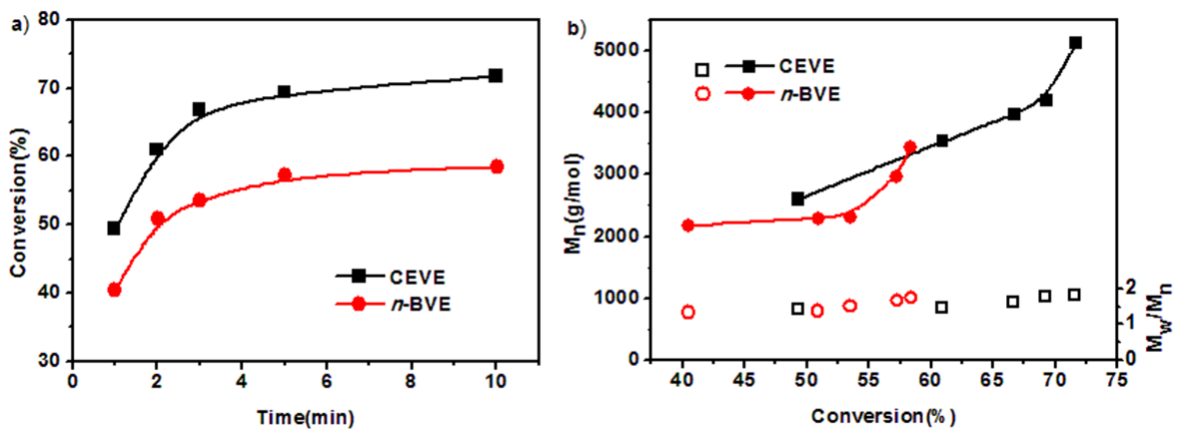
Cationic polymerization of CEVE and *n*-BVE initiated by CumOH/B(C_6_F_5_)_3_/Et_2_O in H_2_O at 20 °C: (**a**) conversion vs time; (**b**) Mn and Mw/Mn vs conversion; [CEVE] = 2.0 M; [*n*-BVE] = 1.6 M; [CumOH] = [B(C_6_F_5_)_3_] = 0.05 M; [Et_2_O] = 0.1 M.

To further understand the vinyl ether polymerization characteristics in suspension, CEVE and *n*-BVE were used as monomers to conduct polymerization. The cationic polymerizations of CEVE and *n*-BVE initiated by CumOH/B(C_6_F_5_)_3_/Et_2_O in aqueous suspensions proceeded in a reproducible manner. The incomplete monomer conversions (<75%) for IBVE (49.32%), CEVE (71.68%), and n-BVE (58.40%) (Figure 3a and Figure 5) cationic polymerizations were observed due to the competition between the propagated consumption of the protonated monomer and the formation of acetaldehyde and other organic compounds, which were analyzed through ^1^H-NMR spectroscopy. [LZ2] The increase of *M_n_* with monomer conversion at the later stage of polymerization was also observed in CEVE and *n*-BVE cationic polymerizations.

### 3.3. Polymerization in Aqueous Emulsions

**Figure 6:**
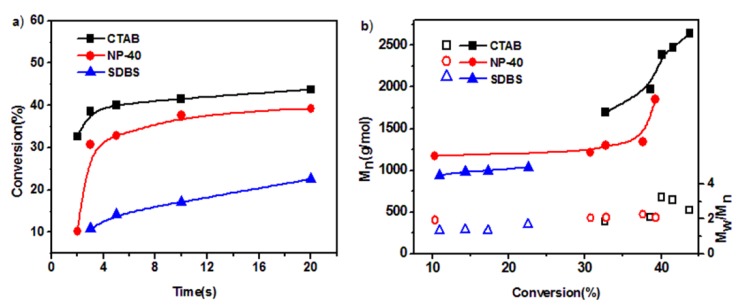
IBVE cationic polymerization initiated by CumOH/B(C_6_F_5_)_3_/Et_2_O in emulsion at 20 °C: (**a**) conversion vs. time; (**b**) *M_n_* and *M_w_*/*M_n_* vs. conversion; [IBVE] = 1.6 M; [CumOH] = [B(C_6_F_5_)_3_] = 0.05 M; [Et_2_O] = 0.1 M; CTAB = 0.02 g; NP-40 = 0.02 g; SDBS = 0.02 g.

**Figure 7:**
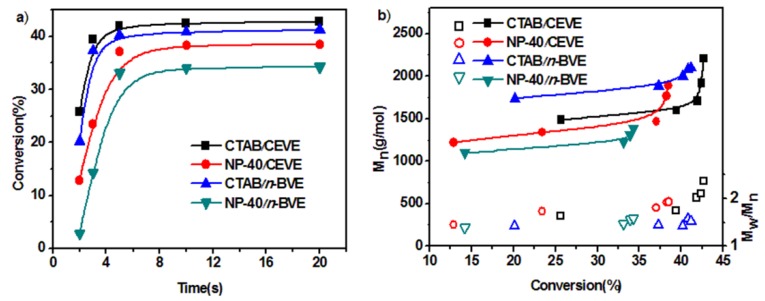
CEVE and *n*-BVE cationic polymerization initiated by CumOH/B(C_6_F_5_)_3_/Et_2_O in an emulsion at 20 °C: (**a**) conversion vs. time; (b) *M_n_* and *M_w_*/*M_n_* vs. conversion; [CEVE] = 2.0 M; [*n*-BVE] = 1.6 M; [CumOH] = [B(C_6_F_5_)_3_] = 0.05 M; [Et_2_O] = 0.1 M; CTAB = 0.02 g; NP-40 = 0.02 g.

To better understand the influence of the aqueous environment on vinyl ether cationic polymerization, poly(IBVE), poly(CEVE), and poly(*n*-BVE) were synthesized in emulsions (using CTAB, SDBS, and NP-40 as cationic, anionic, and non-ionic surfactants, respectively). Only trace amounts of poly(CEVE)s and poly(*n*-BVE)s were synthesized in the emulsion with SDBS at 20 °C. Compared with the polymerizations implemented in H_2_O, polymers with lower monomer conversion and *M_n_* were obtained in the emulsion at the same reaction time (Figure 6 and Figure 7). The emulsion polymerization was also not controlled. These observations are different from those of aqueous cationic polymerization using LASC as a catalyst in previous studies [26,27,28,29]. In these studies, a compound made of a lanthanide triflate and a surfactant, that is, a LASC catalyst, transferred the polymerization sites from the monomer/water surface to the interior of the monomer, resulting in the production of long polymer chains. By contrast, the lower yields were observed during emulsion polymerization, probably because the space steric hindrance of these surfactants on the monomer/water surface placed restrictions on the contact between B(C_6_F_5_)_3_/Et_2_O and the monomer [45]. The lower molecular weights were due to the formation of smaller droplets (average 51–59 nm) in case of emulsion polymerization. Consequently, the polymerization sites were still on the monomer/water surface. The reaction exotherms decreased, and the preparation times during polymerizations increased in the emulsion processes in comparison with those in the high-purity water (Figure S3). In the emulsion with SDBS as an anionic surfactant, mass anions inhibited the positive charge of the active species on the monomer/water surface [45]. Although NP-40 and CTAB are non-ionic and cationic surfactants, the space steric hindrance still suppressed the monomer conversion.

### 3.4. Polymer Characterization

**Figure 8:**
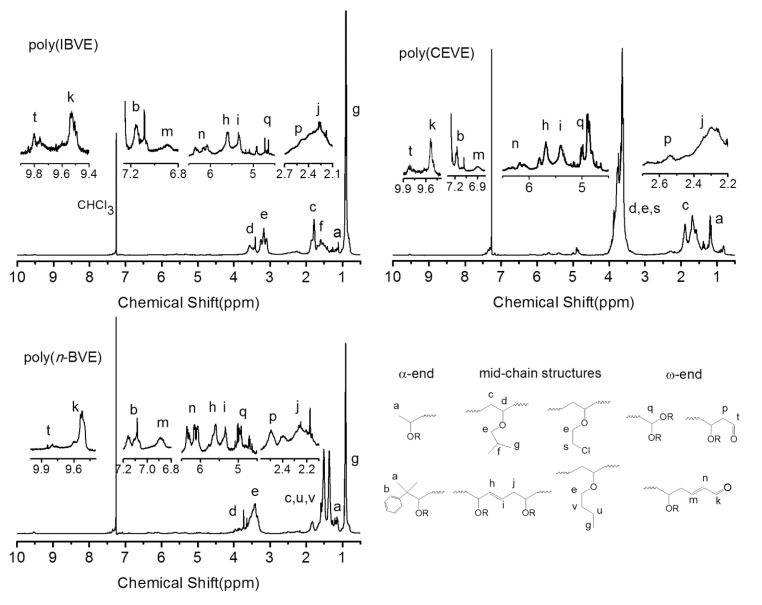
^1^H-NMR spectra of poly(IBVE), poly(CEVE), and poly(*n*-BVE) initiated by CumOH/B(C_6_F_5_)_3_/Et_2_O in aqueous media.

The terminal structures of poly(IBVE), poly(CEVE), and poly(*n*-BVE) obtained in aqueous suspensions and emulsions were examined by ^1^H-NMR spectroscopy (Figure 8). The characteristic signals at 0.90 (peak g), 1.60 (peak f), 1.79 (peak c), 3.17 (peak e), and 3.56 (peak d) ppm were assigned to the main chain protons of the monomer unit of poly(IBVE). The characteristic signals at 1.00–2.09 (peak c) and 3.4–4.00 (peaks d, e, s) ppm were assigned to the main chain protons of the monomer unit of poly(CEVE). The characteristic signals at 0.90 (peak g), 1.10–1.75 (peaks c, u, v), and 3.25–4.00 (peaks d, e) ppm were assigned to the main chain protons of the monomer unit of poly(*n*-BVE). The signals at 1.11 (peak a) and 7.00–7.20 (peak b) ppm were assigned to –CH_3_ and the phenyl group at the α-end (Figure 8), respectively. The signals at 2.52 (peak j), 5.35 (peak i), and 5.60 (peak h) ppm were attributed to the mid-chain (internal) olefin group formed due to chain transfer to the polymer [22]. In addition, the following three types of ω-end groups were examined in the ^1^H-NMR spectra (Figure 8): acetal (resonance at 4.73 ppm (peak q)), aldehyde (resonances at 2.49 (peak p), and 9.81 (peak t) ppm, respectively) and alkenal (resonances at 6.09 (peak n), 6.90 (peak m), and 9.54 (peak k) ppm, respectively).

The content of the ω-end and mid-chain (internal) olefin groups of polymers initiated by CumOH/B(C_6_F_5_)_3_/Et_2_O in aqueous media is presented in Table 3. For polymerization in the emulsion, the content of mid-chain olefin groups (0.1–0.2 double bonds per polymer chain) was present in minor amounts. β-H elimination, a typical chain-transfer reaction for conventional cationic polymerization of vinyl ethers [16,47] leading terminal double-bond groups, was not observed in the ^1^H-NMR spectra of the polymers synthesized in aqueous media. A possible reason was that these reactive terminal double-bond groups were consumed in chain coupling reactions [37], which is one reason the total content of the ω-end groups was lower than 100% (Table 3). Another reason is that a small extent of polymerization was initiated by H_2_O/B(C_6_F_5_)_3_/Et_2_O. The chain coupling reaction led to an overestimation of the phenyl head group content (see Section 3.6 for details).

### 3.5. Possible Interactions Between the Initiator and Co-initiator

**Figure 9:**
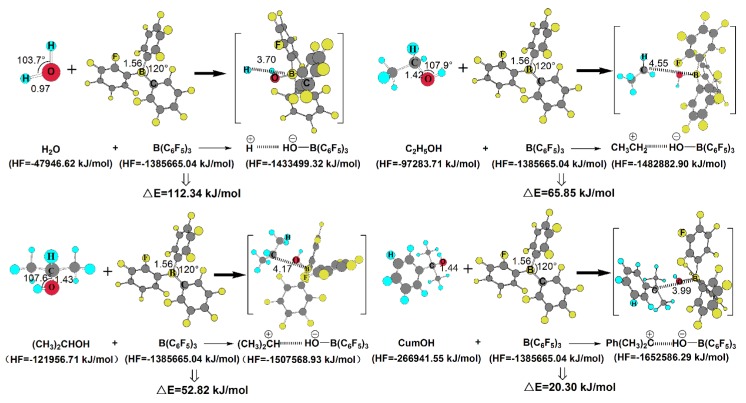
The optimized conformations of H^+^–B(C_6_F_5_)_3_–OH^−^, CH_3_CH_2_^+^–B(C_6_F_5_)_3_–OH^−^, (CH_3_)_2_CH^+^–B(C_6_F_5_)_3_–OH^−^ and (CH_3_)_2_C(C_6_H_5_)^+^–B(C_6_F_5_)_3_–OH^−^ at the 6-31G* level.

Given that H_2_O, EtOH, IPA, and CumOH could induce the aqueous cationic polymerization of vinyl ethers, DFT was applied to investigate the competition between H_2_O and alcohol combined with B(C_6_F_5_)_3_ for providing a theoretical basis. Models of active center were built at the 6-31G* level, such as H^+^–B(C_6_F_5_)_3_–OH^−^, CH_3_CH_2_^+^–B(C_6_F_5_)_3_–OH^−^, (CH_3_)_2_CH^+^–B(C_6_F_5_)_3_–OH^−^, and (CH_3_)_2_C(C_6_H_5_)^+^–B(C_6_F_5_)_3_–OH^−^. The strain energy was minimized through structural optimization in bond angles and lengths. The activation energy required to form H^+^–B(C_6_F_5_)_3_–OH^−^ active center was the most in all these four ion pair models (Figure 9), which indicated that B(C_6_F_5_)_3_ was more readily formed in an active center with alcohols when alcohols were present in the aqueous media. The C^+…^B distance in (CH_3_)_2_C(C_6_H_5_)^+^–B(C_6_F_5_)_3_–OH^−^ (3.99 Å) was the shortest in all the models of active center formed by alcohols and B(C_6_F_5_)_3_. Furthermore, the models of (CH_3_)_2_C(C_6_H_5_)^+^–B(C_6_F_5_)_3_–OH^−^ presented a large steric hindrance, indicating that the propagating carbocation with the CumOH/B(C_6_F_5_)_3_ initiating system was much more sterically hindered, such that more restriction occurred in the direction of the insertion of monomer molecules into the propagating carbocation. Therefore, the polymerization initiated by CumOH/B(C_6_F_5_)_3_ was stable.

### 3.6. Proposed Mechanism for Polymerization

The polymerization (initiation, propagation, and termination) sites were on the monomer/water surface for the aqueous cationic polymerization of vinyl ether initiated by B(C_6_F_5_)_3_/Et_2_O, similar to the aqueous cationic polymerization of styrene derivatives [45,48,49]. However, the polymerization processes shared three special features in comparison with the cationic polymerization of other monomers initiated by other co-initiators. The first feature was the severely exothermic nature and poor reproducibility of vinyl ether polymerization due to the high reactivity of this monomer. To overcome this disadvantage, different initiators, co-solvents (*n*-hexane and toluene), NaCl solutions, and surfactants were added to the experiments. CumOH/B(C_6_F_5_)_3_/Et_2_O induced polymerization in a reproducible manner in the suspension and emulsion. The second feature was about the decrease in monomer conversion and *M_n_* when the reaction temperature was reduced using B(C_6_F_5_)_3_ as a co-initiator; this process was contrary to traditional cationic polymerizations [1,2,3,46]. The third feature was that low yields and *M_n_* were obtained in *n*-hexane/H_2_O, toluene/H_2_O, NaCl solution, and emulsion in comparison with high-purity water; this process was opposite to the aqueous cationic polymerizations using other co-initiators [26,27,28,37].

**Scheme 1:**
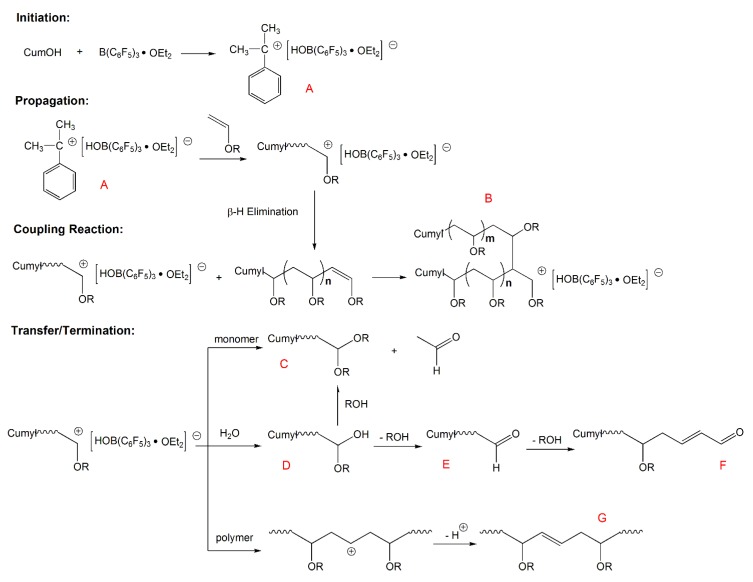
Proposed mechanism for the aqueous cationic polymerization of vinyl ethers using CumOH/B(C_6_F_5_)_3_/Et_2_O.

According to the experimental data, a mechanism for the aqueous cationic polymerization of vinyl ether was proposed (Scheme 1). In the initiation, B(C_6_F_5_)_3_/Et_2_O captured a hydroxyl group from CumOH to form the (CH_3_)_2_C(C_6_H_5_)^+^–Et_2_OB(C_6_F_5_)_3_–OH^−^ active center (structure A). The structure A then initiated the polymerization, and the propagation was extremely fast until chain-transfer reactions or termination occurred. The chain-transfers via β-H elimination formed a terminal double-bond group that was highly reactive [37,50]. This terminal olefin group readily reacted with structure A, thereby forming coupled chains that resulted in polymers with two head groups (structure B) [35,51]. The chain-transfers by the monomer generated an acetal end group (structure C) [22]. The chain-transfers by water generated an hydroxyl end group (structure D), but the low stability of hemiacetal for poly(vinyl ether)s led to hydroxyl end group (structure D) conversion into an aldehyde end group (structure E) or acetal end group (structure C). The aldehyde end group lost ROH to form an alkenal end group (structure F) [37]. In addition, only trace amounts (Table 3) of mid-chain olefin groups (structure G) were detected in the aqueous polymerization due to chain-transfer to the polymer [35], which was a typical side reaction in the conventional cationic polymerization of vinyl ethers.

## 4. Conclusions

Poly(IBVE)s, poly(CEVE)s, and poly(*n*-BVE)s were successfully achieved in aqueous suspensions and emulsions using B(C_6_F_5_)_3_/Et_2_O as a co-initiator and alcohols as an initiator in a reproducible manner. Polymerization characteristics were systematically tested and compared in the suspension and emulsion. Adding co-solvents (*n*-hexane and toluene), NaCl, and surfactants could transfer heat effectively in the reaction. However, low yields and molecular weights were obtained in comparison with high-purity water. The polymerization rate surprisingly decreased when the temperature was lowered; this result was opposite to that in traditional cationic polymerizations. The aqueous cationic polymerization sites were located on the monomer/water surface. The end group structures of polymers examined by ^1^H-NMR spectroscopy indicated that various chain-transfer reactions (to monomers, water, and polymers) occurred in these processes. Accordingly, the mechanism for the aqueous cationic polymerization of vinyl ethers using CumOH/B(C_6_F_5_)_3_/Et_2_O was proposed.

## Figures and Tables

**Table 1:** Suspension cationic polymerization of isobutyl vinyl ether (IBVE) in the presence of different initiators ^a.^

run	Initiator (content) (mmol)	Conv.^b^ (±S) (%)	*M_n_^c^* (GPC) (g mol^−1^)	*M_w_/M_n_* ^c^
1	H_2_O (∞)	30.1 (±15.4)	4690	1.88
2	EtOH (0.25)	45.2 (±15.6)	4310	2.01
3	IPA (0.25)	43.3 (±7.7)	5940	1.57
4	CumOH (0.25)	48.3 (±1.6)	6270	1.39

^a^ Polymerization conditions: H_2_O (total): 5 mL; IBVE: 1 mL; B(C_6_F_5_)_3_: 0.25 mmol; *T* = 20 °C; mechanically stirring at 150 rpm. ^b^ Conversion value came from the average of 5 experimental runs with each initiator along with the respective standard deviation. ^c^ *M_n_* and *M_w_/M_n_* value came from the polymer of which conversion was closest to the averages of each group.

**Table 2:** Suspension cationic polymerization of IBVE at different temperatures ^a^.

Run	Medium	T (°C)	Time (min)	Conv. (%)	*M_n_* (GPC) (g mol^−1^)	*M_w_/M_n_*
5	H_2_O	20	5	48.9	6500	1.39
6	H_2_O	0	10	27.4	5020	1.41
7	*n*-hexane/H_2_O	20	17	44.2	4810	1.32
8	*n*-hexane/H_2_O	0	21	40.7	4690	1.28
9	*n*-hexane/H_2_O	−5	27	24.7	4410	1.36
10	Toluene/H_2_O	20	17	42.6	4610	1.37
11	Toluene/H_2_O	0	21	35.1	4550	1.40
12	Toluene/H_2_O	−5	27	22.7	4090	1.32
13	NaCl solution ^b^	20	15	37.3	4800	1.36
14	NaCl solution ^b^	0	27	28.7	3040	1.42
15	NaCl solution ^b^	−10	47	23.0	2120	1.31

^a^ Polymerization conditions: H_2_O (total): 5 mL; *n*-hexane: 1 mL; toluene: 1 mL; IBVE: 1 mL; B(C_6_F_5_)_3_: 0.25 mmol; CumOH: 0.25 mmol; mechanically stirring at 150 rpm. ^b^ NaCl: 1 g.

**Table 3:** Functionality at the ω-end and mid-chain (internal) olefin group content of polymers initiated by CumOH/B(C_6_F_5_)_3_ at 20 °C in aqueous media.^a^

run	Media	Acetal ^b^ (%)	Aldehyde ^b^ (%)	Alkenal ^b^ (%)	Internal olefin ^c^ (per chain)
16	H_2_O	31.8	31.5	26.7	0.4
17	*n*-hexane/H_2_O	28.2	35.1	28.7	0.3
18	Toluene/H_2_O	27.0	36.0	27.0	0.4
19	NaCl solution	17.0	46.1	30.0	0.5
20	CTAB	27.9	39.6	23.5	0.1
21	NP-40	28.8	33.4	27.9	0.2
22	SDBS	39.8	34.8	19.5	0.1
23 ^d^	H_2_O	33.8	20.6	3.2	0.4
24 ^e^	H_2_O	11.7	41.6	38.6	0.8

^a^ IBVE Polymerization conditions, see Table 2. ^b^ Calculated by ^1^H-NMR spectroscopy as acetal [5*I*(q)/*I*(b)], aldehyde [5*I*(t)/*I*(b)], and alkenal [5*I*(k)/*I*(b)]. ^c^ Calculated as the ratio of integrals (normalized to one hydrogen) as 5*I*(i)/*I*(b). ^d^ CEVE polymerization, [CEVE] = 2.0M. ^e^ *n*-BVE polymerization, [*n*-BVE] = 1.6 M.

## Supplementary Materials

The following are available online at https://www.mdpi.com/2073-4360/11/3/500/s1, Figure S1: Suspension polymerization of styrene at 20 °C: (a) conversion vs time; (b) ln[M_0_]/[M] vs time. [St] = 1.75 M; [B(C_6_F_5_)_3_] = 0.05 M.; Figure S2: Styrene conversion at different polymerization temperatures initiated by CumOH/B(C_6_F_5_)_3_ in aqueous suspension for 50 h. [St] = 1.75 M; [CumOH] = [B(C_6_F_5_)_3_] = 0.05 M; NaCl: 1 g.; and Figure S3: Temperature during cationic polymerizations initiated by CumOH/B(C_6_F_5_)_3_ in aqueous emulsion at 20 °C. [IBVE] = 1.6 M; [CEVE] = 2.0 M; [n-BVE] = 1.6 M; [CumOH] = [B(C_6_F_5_)_3_] = 0.05 M; CTAB = 0.02 g; NP-40 = 0.02 g; SDBS = 0.02 g.
