# Supplementary material for: Characteristics and Mechanism of Vinyl Ether Cationic Polymerization in Aqueous Media Initiated by Alcohol/B(C6F5)3/Et2O
Source: Polymers (Basel). 2019 Mar 14;11(3):500. doi: 10.3390/polym11030500 (PMC6473351; doi:10.3390/polym11030500)
Supplement: Supplementary file 1 [file polymers-11-00500-s001.pdf]

Characteristics and Mechanism of Vinyl Ether Cationic Polymerization in Aqueous Media Initiated by Alcohol/$B(C_6F_5)_3$/Et_2O

Jinghan Zhang[1,3], Yibo Wu[2,3]*, Kaixuan Chen[2], Min Zhang[2,3], Liangfa Gong[2], Dan Yang[2,3], Shuxin Li[2,3], Wenli Guo[1,3]*

[a]College of Material Science and Engineering, Beijing University of Chemical Technology, Beijing 100029, China.

[b]Department of Materials Science and Engineering, Beijing Institute of Petrochemical Technology, Beijing, 102617, China.

[c]Beijing Key Lab of Special Elastomeric Composite Materials, Beijing, 102617, China.

*Corresponding author: Yibo Wu, Beijing Institute of Petrochemical Technology, Beijing, 102617, China, E-mail: wuyibo@bipt.edu.cn

Wenli Guo, Beijing University of Chemical Technology, Beijing 100029, China, E-mail: gwenli@bjzx.gov.cn

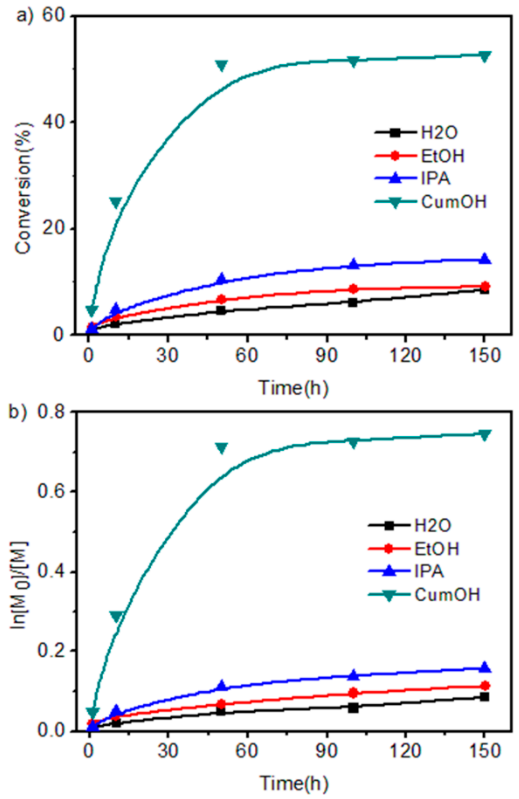

Figure S1. Suspension polymerization of styrene at 20 °C: (a) conversion vs time; (b) $\ln[M_0]/[M]$ vs time. [St] = 1.75 M; $[B(C_6F_5)_3]$ = 0.05 M.

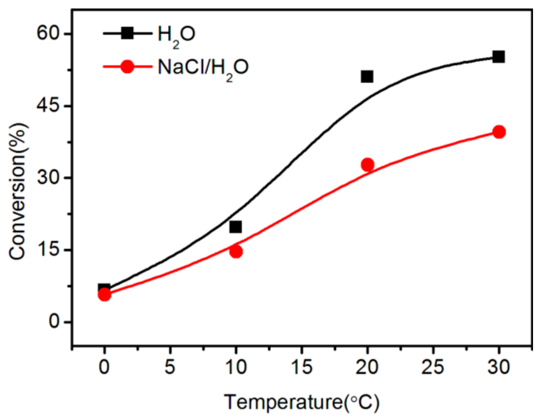

Figure S2. Styrene conversion at different polymerization temperatures initiated by CumOH/$B(C_6F_5)_3$ in aqueous suspension for 50 h. [St] = 1.75 M; [CumOH] = $[B(C_6F_5)_3]$ = 0.05 M; NaCl: 1 g.

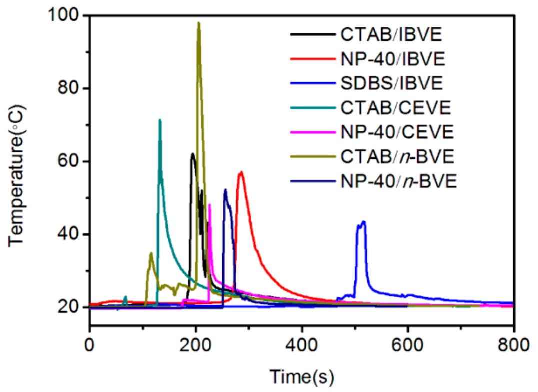

Figure S3. Temperature during cationic polymerizations initiated by CumOH/B(C_6F_5)$_3$ in aqueous emulsion at 20 °C. [IBVE] = 1.6M; [CEVE] = 2.0 M; [n-BVE] = 1.6 M; [CumOH] = [B(C_6F_5)$_3$] = 0.05 M; CTAB = 0.02 g; NP-40 = 0.02 g; SDBS = 0.02 g.
